# Development of an open-source 3D imaging method for forensic age estimation based on medial clavicular ossification: assessing area and volume ratios of epiphyses and metaphyses

**DOI:** 10.1007/s00414-025-03614-y

**Published:** 2025-09-27

**Authors:** Jonathan Kurz, Tobias Krähling, Ronald Schulz, Christian Ottow, Volker Vieth, Andreas Schmeling, Aaron Liebsch

**Affiliations:** 1 Institute of Legal Medicine, University of Münster and University Hospital Münster, Röntgenstraße 23, 48149 Münster, Germany; 2 Clinic for Radiology, University of Münster and University Hospital Münster, Albert- Schweitzer-Campus 1, 48149 Münster, Germany; 3 Clinic for Radiology, Klinikum Ibbenbüren, Große Straße 41, 49477 Ibbenbüren, Germany

**Keywords:** Forensic age estimation, Computed tomography, Medial clavicular epiphysis, Norm variants, 3D model, Morphometric analysis

## Abstract

Forensic age estimation is essential for legal and social decision-making when reliable documentation is lacking. Traditionally, ossification of the medial clavicular epiphysis (MCE) is assessed by visual staging, but norm variants frequently limit classic systems and introduce error and irreproducibility. High-resolution computed tomography (CT) allows for quantitative morphometric assessment, potentially offering support – especially in such cases. Based on the approach of Hua et al. (2014) an open-source workflow for metric age estimation of the medial clavicles using semi-automatic three-dimensional (3D) CT segmentation was developed. Clinical CT scans were pseudonymized, archived in XNAT (Extensible Neuroimaging Archive Toolkit), and 3D models were generated in 3D Slicer. Expert-guided segmentation and alignment enabled extraction of quantitative parameters including planar areas and volumes of epiphyses and metaphyses; area and volume ratios were calculated as dimensionless metrics. It was concluded that morphometric assessment of the medial clavicles via 3D imaging is a promising approach for forensic age estimation. The workflow’s open-source architecture supports transparency and collaborative validation. Future research should validate metric markers and pursue workflow automation, particularly to address anatomically complex cases.

## Introduction

Reliable forensic age estimation in living individuals is of high importance in legal, immigration, and social contexts, in cases where identity papers are lacking or doubts arise regarding the stated chronological age [1–3]. In Germany and many other countries, legal thresholds at ages 14, 18, and 21 are of particular relevance for criminal, asylum and civil proceedings [2, 4]. In age assessment practice, only computed tomography (CT) examination of the clavicles currently allows for proof beyond reasonable doubt that the ages of 18 and 21 have been completed [3, 5]. With the advent of high-resolution thin-slice CT, the accuracy and reproducibility of age estimation based on the assessment of the medial clavicular epiphysis (MCE) have improved considerably [6–8]. Staging systems for MCE ossification, in particular the 5-main-stage system of Schmeling et al. [9, 10] and the additional 6-substage system of Kellinghaus et al. [7], provide the scientific basis for standardized assessment, and increase precision, especially at critical legal boundaries.

However, MCE-based age estimation is complicated by marked anatomical variability, referred to as norm or shape variants, which can be divided into three main types: Concavities of the metaphysis (sometimes referred to as “bowl” or “fish-mouth” forms for instance), multiple epiphyseal ossification centers and other irregular forms [11]. These morphologies often preclude a clear assignment to classical stages and are a frequent source of error, especially for less experienced examiners [12]. The need for expert knowledge about the entire spectrum of these norm variants is recognized as mandatory for qualified casework [11, 12]. The comprehensive CT-Atlas by Rudolf et al. [13] systematically catalogues this morphological diversity in a sample of 3,041 sternoclavicular CT scans, demonstrating that 10–20% of medial clavicles exhibit such norm variants, a finding also mirrored in other cohorts [14, 15]. As the correlation between development of these norm variants and age may differ, they are currently explicit excluded from forensic age estimation. Accordingly, the currently used method of age estimation by stages requires a very high level of expertise that might not be available everywhere. But even if this condition is met, age estimation is not possible in cases of norm variants.

In addition to the classic staging systems, metric approaches using CT-derived morphometric parameters have been introduced, which aims to offer objective, quantitative alternatives or supplements to visual staging, particularly in ambiguous or anatomically atypical cases. Hua et al. reported a correlation between age and epiphyseal/metaphyseal diameter and area ratios [16]. The present study aims to develop an open-source morphometric approach for forensic age estimation of the medial clavicles based on that approach of Hua et al. The three-dimensional (3D) imaging and morphometric analysis integrates expert-validated staging.

## Methodological proposal

### Sample collection and processing

For this project, CT images were provided by the Clinic for Radiology at University Hospital Münster. In the Radiological Information System (RIS), all cases in which images of the medial end of the clavicles were likely included as part of the CT scan were identified. Only Siemens CT scanners were used, mostly we chose trauma CT studies on the 64-slice scanner Siemens Somatom Definition AS+. Typical scan and dose parameters are as follows: tube potential: 120 kVp; tube current-time product: 251.8 ±95.8 mAs (mean ±SD); automatic mA modulation; spiral pitch factor p: 0.8; CT dose index CTDI_vol_: 16.79 ±6.31 mGy (mean ±SD), convolution kernel: B70f. Overall, the range of slice thickness was 0.6–1.5 mm (min. – max.). Prior to analysis, patient data were pseudonymized. All CT images were then transferred to an XNAT (Extensible Neuroimaging Archive Toolkit) server, XNAT is an open-source imaging informatics platform commonly used in medical research [17, 18]. One advantage of the XNAT solution is that the results and all intermediate steps from 3D Slicer (see next section for details) can be saved back to the XNAT server as additional data for the respective case, which makes it easy to assign the data. In addition, by using the server to store all image and evaluation data, there is a central storage location, allowing several investigators to work on the evaluation of the data sets in parallel. Only http(s) access to the XNAT server is required from the computer on which 3D Slicer is running.

### Producing a 3D model in 3D slicer

To obtain metric measurements, a 3D model of the medial clavicle for each side, including the epiphyseal ossification center(s), was generated using the open-source software 3D Slicer (version 5.6.2) [19]. The CT data were imported into 3D Slicer via the “XNATSlicer” extension. Initially, the region of interest (ROI) was manually cropped from the CT dataset using the “Crop Volume” module in 3D Slicer. Subsequently, segmentation of the ROI was performed semi-automatically with the “Segment Editor.” The threshold range was set from a minimum of 300 Hounsfield units (HU) to the maximum possible value. This threshold was applied for masking, and the “Paint” function of the segment editor was used to annotate the ROI on multiple slices, ensuring accurate segmentation of both bone and ossification center(s). Further refinement of the segmentation was accomplished using the “Grow from Seeds,” “Wrap Solidify,” and “Logical Operators” functions within the segment editor. After segmentation, 3D Slicer generated the corresponding 3D model.

### Area measurement

**Fig. 1:**
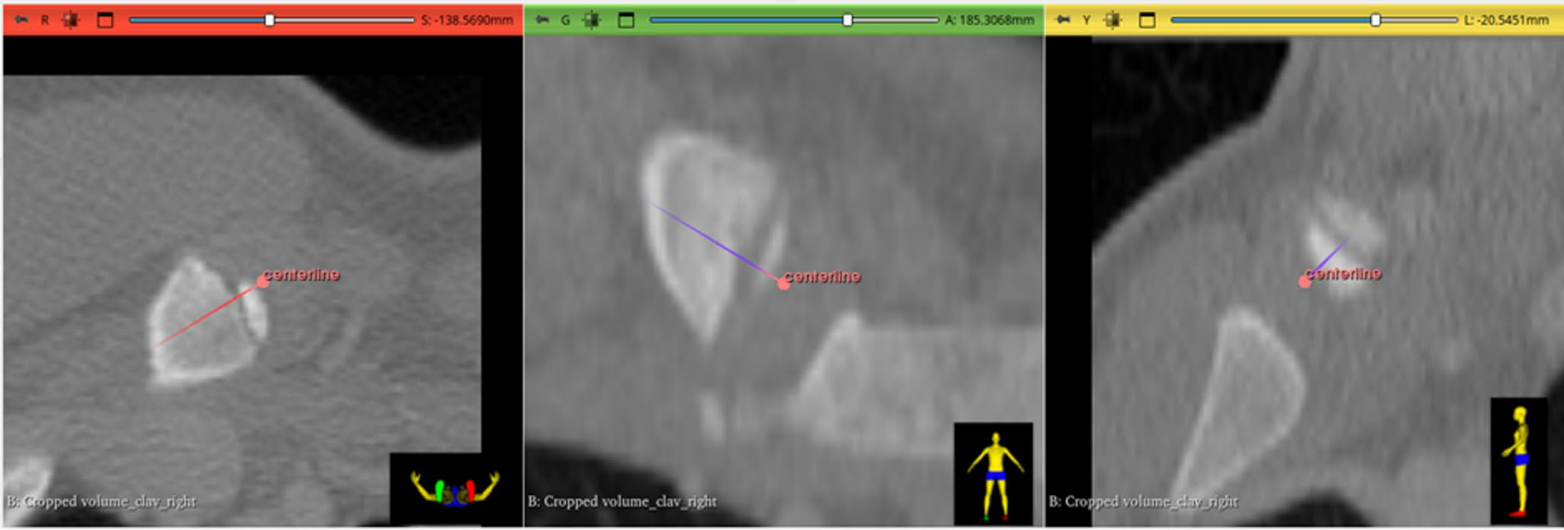
For precise alignment of the 3D model, to obtain an optimal en-face view of the proximal end of the medial clavicle, a centerline was drawn in the CT image. The central position of the proximal endpoint of the centerline anterior to the epiphysis was verified in all three CT image planes. (red: axial view, green: coronal view, yellow: sagittal view)

**Fig. 2:**
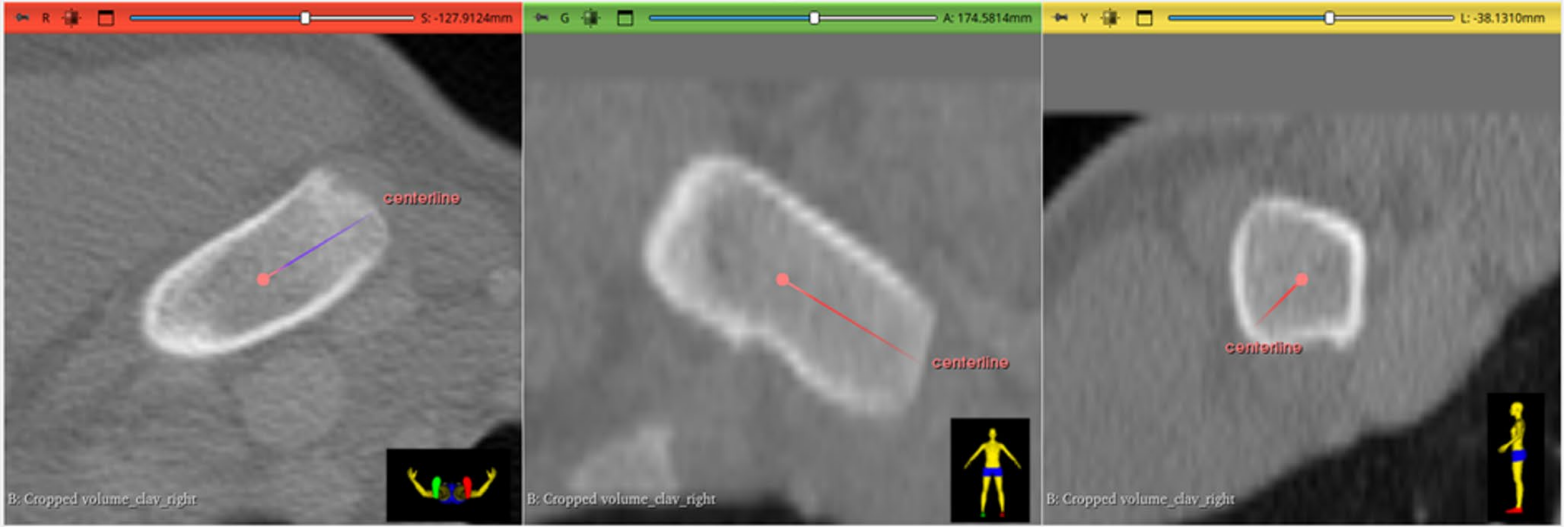
The central position of the distal endpoint of the centerline within the metaphysis was verified in all three CT image planes. (red: axial view, green: coronal view, yellow: sagittal view)

**Fig. 3:**
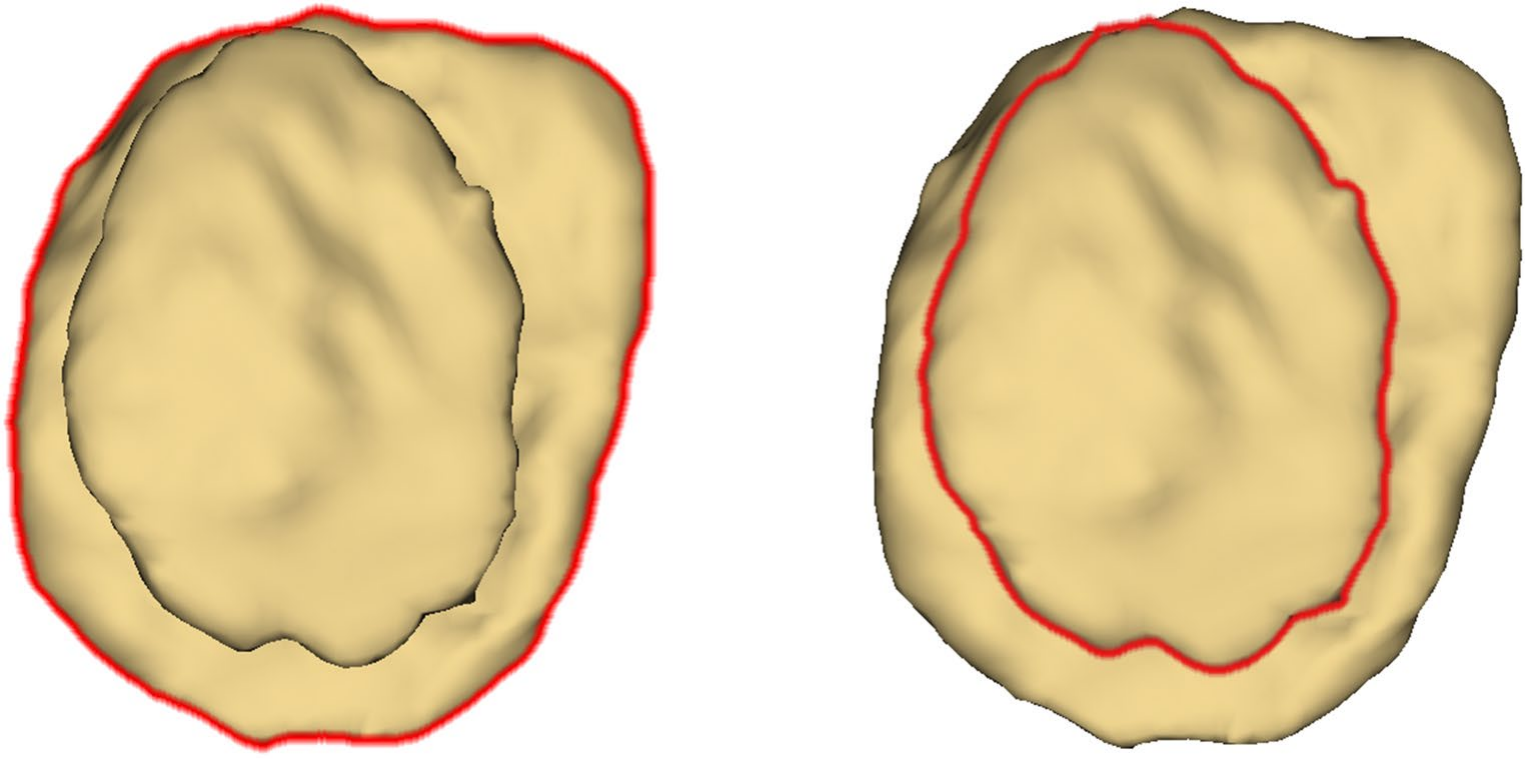
En-face view onto the proximal end of the medial clavicle. Left: outline of the metaphysis. Right: outline of the epiphysis. Outlines were automatically selected by the “Lasso Tool” in Photoshop

For measurement of the areas of the epiphysis and metaphysis, it was necessary to align the 3D model to obtain an en-face view of the proximal end of the medial clavicle. To ensure precise alignment, a centerline was drawn on the original CT images, with the proximal endpoint placed anterior to the epiphysis and the distal endpoint positioned within the metaphysis. The central position of both endpoints was verified in all three CT imaging planes (see Figs. 1 and 2). If the line appeared as a single point, rather than a line, in the 3D model, optimal en-face orientation was confirmed. A screenshot of this view was then taken, and the flat areas of both the epiphysis and metaphysis were determined by pixel count using Adobe Photoshop. For automated selection of respective areas, the “Lasso Tool” in Photoshop was used. The two area measurements (in pixels) were then compared, and a dimensionless ratio was created by dividing the area of the epiphysis by the area of the metaphysis. Figure 3 visualizes the analysis process. When the measurements were repeated after realignment a certain deviation in the results was found. Area determination and pixel counting can be equally performed in open-source software such as ImageJ, using manual selection tools like the built-in “Freehand Selection” or the plugin-based “Lasso Selection Tool”, as well as automated methods such as “Thresholding” combined with “Analyze Particles”.

### Volume measurement

**Fig. 4:**
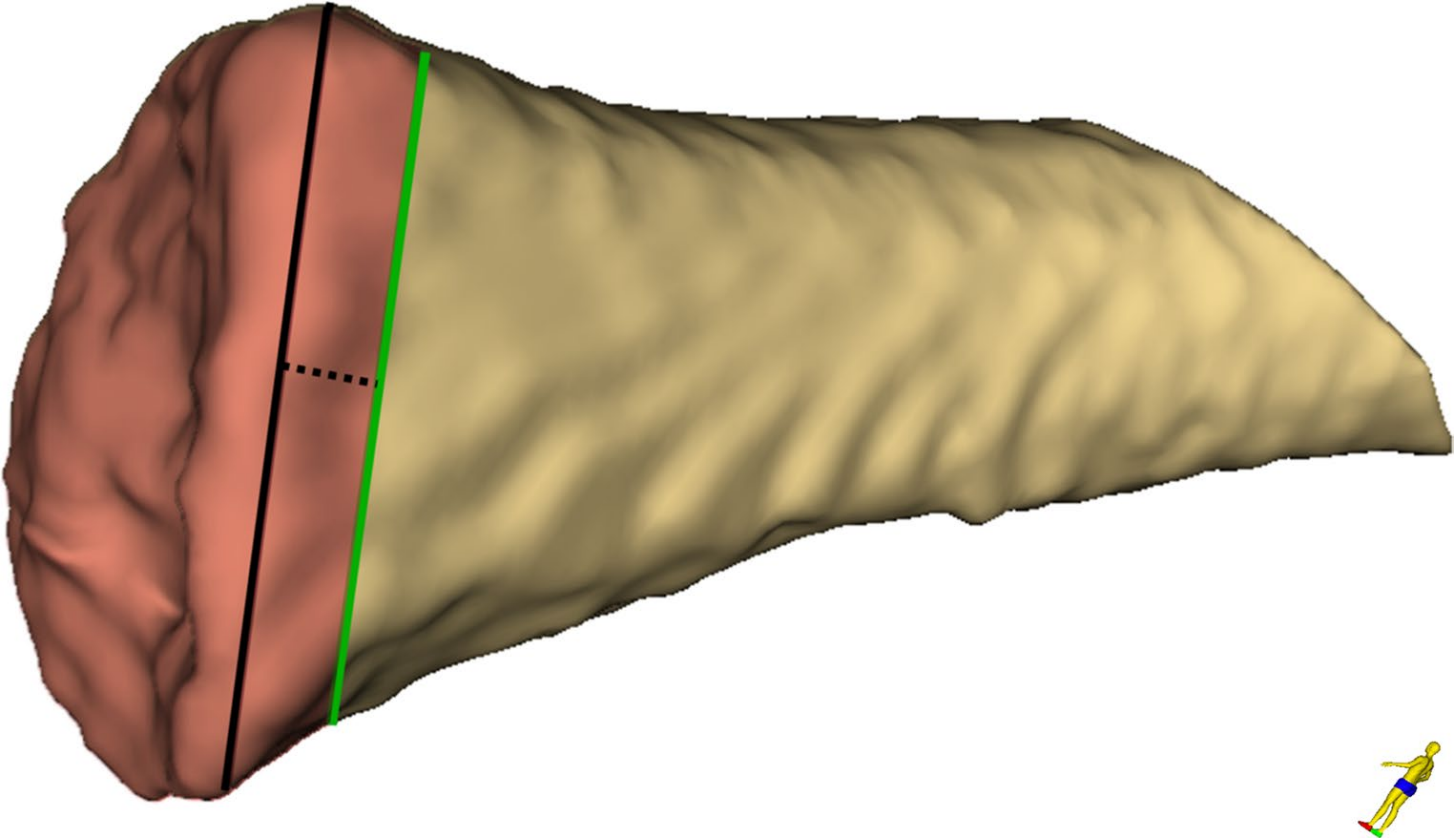
Schematic visualization of the extent of the metaphysis of a right clavicle. The black line indicates the greatest diameter of the metaphysis; the dotted line represents one third of the radius of the black line (reference value) and the green line indicates the distal end of the measurement

**Fig. 5:**
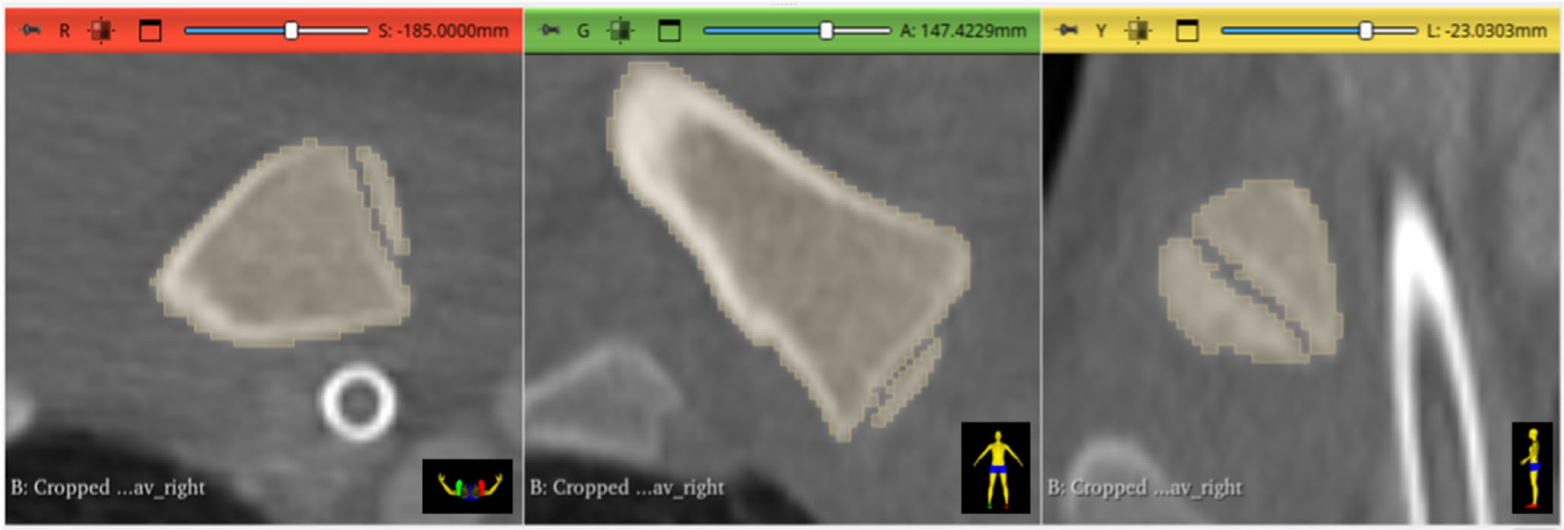
Screenshot of a fully segmented medial clavicle in all three imaging planes. (red: axial view, green: coronal view, yellow: sagittal view)

**Fig. 6:**
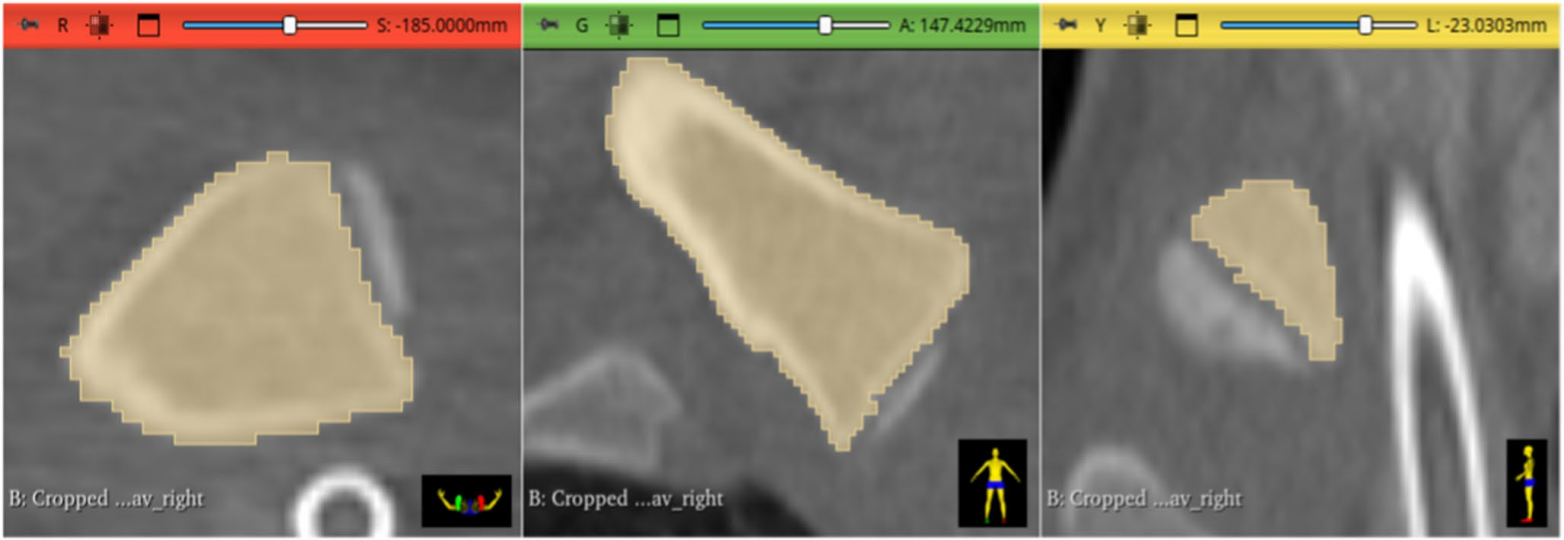
Screenshot of a segmentation after removal of the epiphyseal ossification center using the “Erase” function of “Segment Editor” in all three imaging planes. (red: axial view, green: coronal view, yellow: sagittal view)

**Fig. 7:**
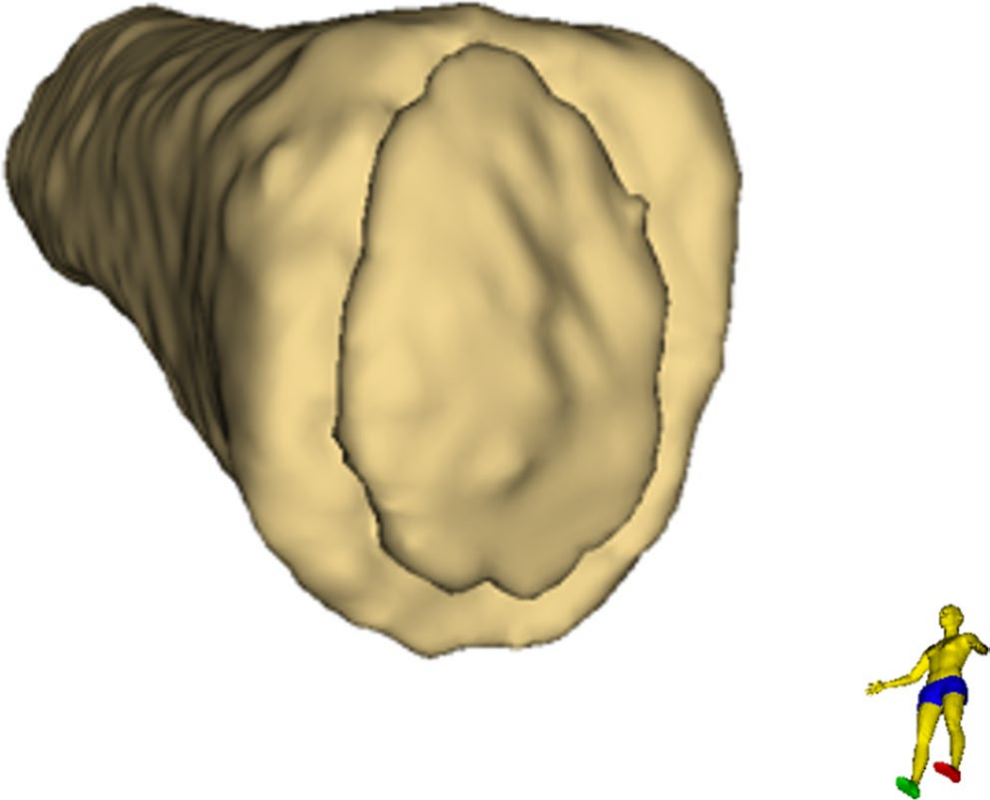
Proximal view onto the 3D reconstruction of a right medial clavicle, including the epiphyseal ossification center, used for the measurement process

To determine the epiphyseal and the metaphyseal volume, the previously generated 3D model was analyzed using the “Segment Cross Section Area” module in 3D Slicer. The actual volume was derived from this module’s output. The distal extent of the metaphysis was defined by the greatest diameter of the metaphysis plus a reference value equivalent to one third of the radius at that position (see Fig. 4). The introduction of an arbitrary value as a relative reference is necessary to compensate for differences arising purely from variations in body size – and the corresponding clavicle dimensions – which could not have been accounted for by setting a constant value for all cases. In the absence of definable anatomical boundaries between metaphysis und diaphysis, the value of one third of the metaphysis’ radius, at the position of its greatest diameter, proved to be heuristically the most robust for the given segmentation settings. This procedure yielded a combined volume of metaphysis and epiphysis (see Fig. 5). For determination of the volume of the epiphysis, ossification center(s), and fusion area (if present) were manually removed from the segmented model using the “Erase” function in the segment editor on the original CT images (see Fig. 6). A second measurement was then performed using the same measurement tool to determine the volume of the isolated metaphysis. The volume of the epiphysis was calculated by subtracting the metaphyseal volume from the initial combined volume of metaphysis and epiphysis. A dimensionless ratio was then created by dividing the volume of the epiphysis by the volume of the metaphysis. Figure 7 shows a typical 3D reconstruction used for the measurement process.

**Fig. 8:**
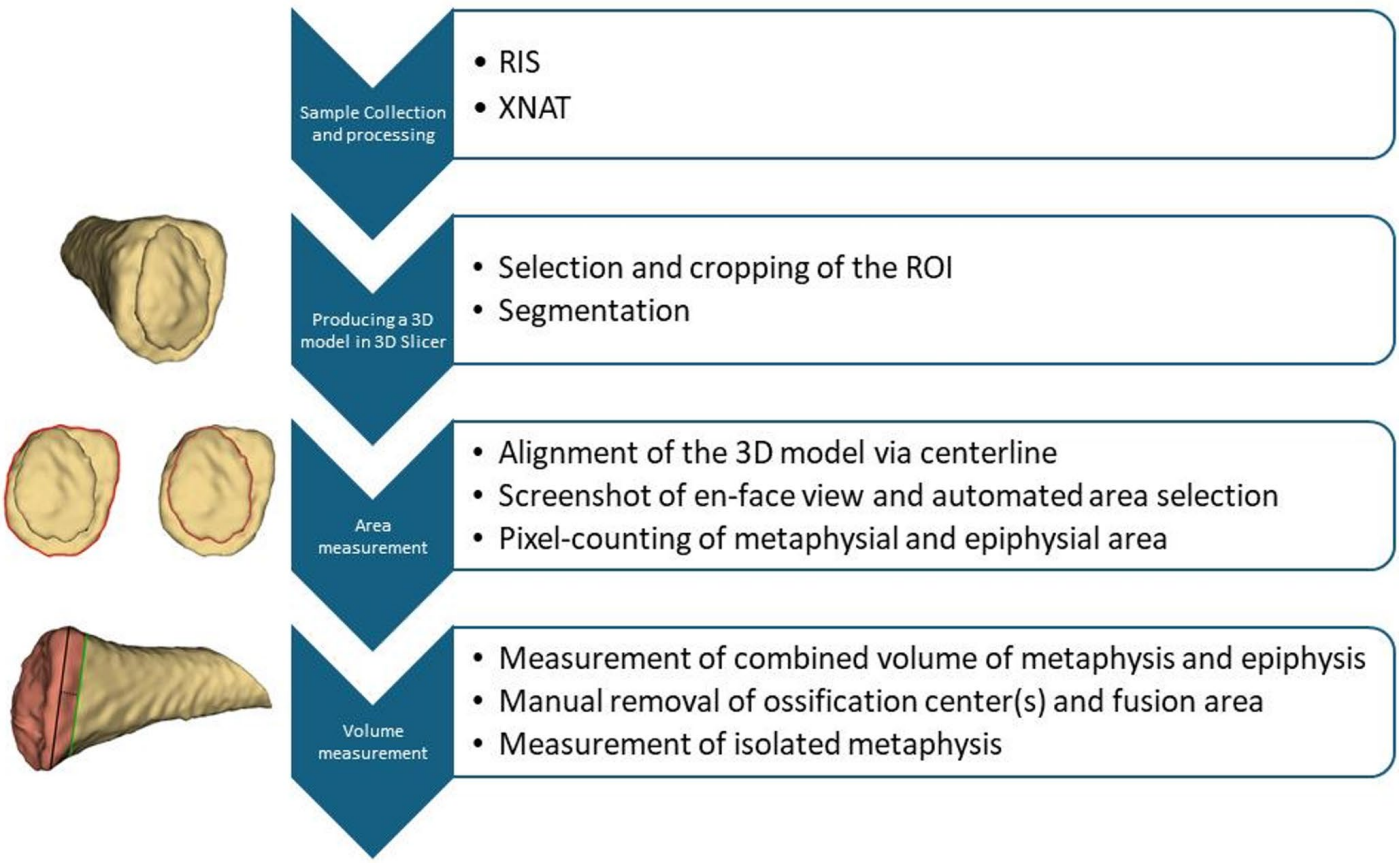
Schematic representation of the established workflow

**Fig. 9:**
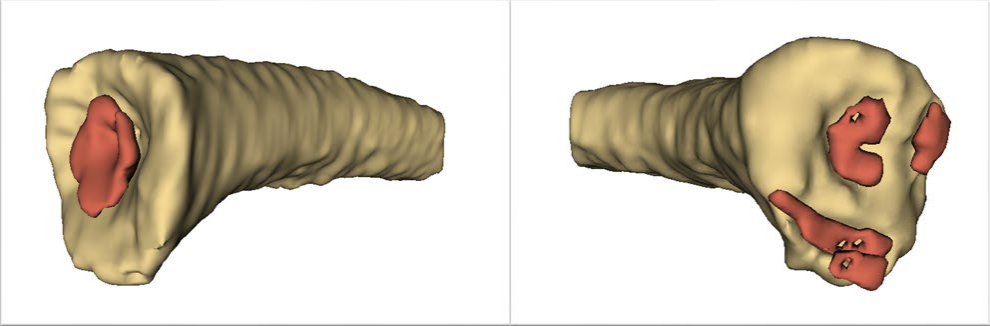
3D reconstruction of medial epiphysis with anatomical norm variants, each of a left clavicle. The ossification centers were colorized in red for better visibility. Left: colorized ossification center embedded in its metaphyseal concavity, right: multiple ossification centers

The established workflow is shown schematically in Fig. 8. It proved applicable for documenting and analyzing anatomical norm variants of the medial clavicle. The segmentation and measurement processes described above could be performed for cases presenting with atypical morphologies such as pronounced metaphyseal concavity or presence of multiple ossification centers. Figure 9 presents two illustrative cases, demonstrating accurate visualization of such norm variants.

## Discussion

The primary objective of this methodological study was to assess the feasibility of morphometric age estimation of the medial clavicle using distances, areas, and volumes based on 3D CT data, following the approach described by Hua et al. [16]. While the original methodology emphasized the use of linear distance measurements, the anatomical definition of precise measuring points for this purpose proved unfeasible due to the lack of clear landmarks and the complex and variable three-dimensional shape of epiphysis and metaphysis, especially in cases exhibiting morphological variants. This finding is congruent with previous literature, which has underscored the influence of anatomical variability on measurement reproducibility [7, 11, 12]. Conversely, both area-based and volumetric analyses emerged as approaches that are more feasible.

A central methodological challenge encountered in this study was the definition of anatomical boundaries. The transition between epiphysis and metaphysis is continuous and lacks discrete demarcations in many cases, complicating efforts to consistently delineate regions for area measurement. Standardized alignment of the clavicle in an en-face orientation was therefore found to be technically demanding due to the subtle curvature and orientation of the bone at the proximal end. Placement of the anatomical centreline involves a potential source of subjectivity. In principle, different positions of the arms among the cases that are possible during the CT imaging process, arms-down or arms-up positon, do not represent a limitation, as it does not alter the process of age estimation itself [20].

Similar to the centerline method, the multi-step segmentation and analysis workflow, notably the process of manually removing the epiphysis for accurate volumetric analysis, introduces an additional potential source of subjectivity. Our observations indicate that small ossification centers and the presence of neighboring or overlapping anatomical structures (e.g., calcified tendons or vascular calcifications) can further complicate segmentation, a finding consistent with prior work highlighting the challenges of imaging in anatomically complex regions [11, 12].

Despite these limitations, 3D imaging techniques can underpin an open-source workflow for the metric assessment of the medial clavicle. The use of fully open-source tools (XNAT and 3D Slicer) enables multi-institutional collaboration.

## Outlook

The method presented here must be applied to a sufficient sample size in order to investigate the correlation between the ratios of the areas and volumes of epiphyses and metaphyses with chronological age. Furthermore, this method should be applied to a comprehensive sample of cases with anatomical norm variants to investigate whether the correlation between the development of these norm variants and chronological age differs from typical cases, which is still unknown. In the future, this approach may offer potential for metric age estimation and could provide solutions in cases where current age estimation methods are not applicable, particularly for norm variants.
